# Supplementary material for: WordCommentsAnalyzer: A windows software tool for qualitative research
Source: F1000Res. 2018 Sep 4;7:536. Originally published 2018 May 3. [Version 2] doi: 10.12688/f1000research.14819.2 (PMC6137411; doi:10.12688/f1000research.14819.2)
Supplement: Supplementary file 3 [file f1000research-7-17614-s0002.tgz › b2a79496-4ada-47c4-9682-a99ce867836d.zip!/Eight Iranian Health Journals/The Journal of Toloo-e-Behdasht_19.docx]

© The Journal of Toloo-e-Behdasht. This work is licensed under CC BY 4.0 (https://creativecommons.org/licenses/by/4.0/).

Volume & Issue: دوره 14 - شماره 2

Title: بررسی میزان تأثیر برنامه آموزشی بر تحرک بدنی خانم‌های خانه دار متأهل 45-20 ساله شهر نور

http://tbj.ssu.ac.ir/article-1-2476-fa.html

زمینه و هدف: بر اساس شاخص‌های مردم تا سال 2010، انجام فعالیت جسمانی منظم به‌عنوان اولین اولویت سبک زندگی سالم عنوان‌شده است. هدف این مطالعه بررسی تأثیر آموزش بر تحرک بدنی خانم‌های خانه‌دار بود. روش بررسی: در این مطالعه  مداخله ای قبل و بعد، تعداد 106 نفر در قالب دو گروه (53 نفر گروه مداخله و 53 نفر گروه کنترل) با روش نمونه‌گیری تصادفی انتخاب شدند. ابزار جمع‌آوری داده‌ها، پرسشنامه بین‌المللی فعالیت فیزیکی بود. پرسشنامه‌ها به‌صورت پیش‌آزمون و پس‌آزمون در دو گروه مداخله و کنترل با فاصله 3 ماه و بعد از آموزش در گروه مداخله تکمیل گردید. آموزش به صورت سخنرانی، پاورپوینت، سی دی آموزشی و پمفلت مرتبط با موضوع، در اختیار گروه مداخله قرار گرفت. یافته‌های پژوهش در پایان، دو گروه مداخله و کنترل وبا استفاده از نرم‌افزار SPSS نسخه 21 مورد تجزیه‌وتحلیل قرار گرفتند. یافته‌ها: در این بررسی زنان متاهل خانه دار در گروه سنی 20 تا 45 سال قرار داشتند. و از تحصیلات ابتدایی تا فوق لیسانس برخوردار بودند. معادل متابولیک کلی فعالیت بدنی در گروه مداخله P=0.74  تفاوت معنی داری مشاهده نشدولی در گروه کنترل P=0.004 تفاوت معنی­داری مشاهده شد.. معادل پیاده­روی کلی در گروه مداخله P=0.048 و در گروه کنترل نیز P=0.023 که معنی­داری در هر دو مشاهده شد . هم چنین تغییرات  معنی­داری در نمره میانگین دو گروه  مورد مطالعه مشاهده شد.P=0.003 با توجه  به نتایج مشاهده شده تأثیر آموزش صر ف در گروه مداخله به تنهایی رد می­شود. نتیجه‌گیری: استفاده از رسانه­های آموزشی مانند سی دی و پمفلت در کنار آموزش با پاورپوینت و وسایل کمک آموزشی مانند پروژکشن و استمرار آن می­تواند در ایجاد آگاهی برای انجام فعالیت­های ورزشی و تحرک بدنی مفید و مثمر ثمر باشد.

Title: بررسی اپیدمیولوژیک موارد مثبت آزمایشات HCV، HBV و HIV در بیماران مورد جراحی و اقدامات تهاجمی قلبی در بیمارستان افشار یزد طی سال های 91-1387

http://tbj.ssu.ac.ir/article-1-1553-fa.html

مقدمه:در بیماران تحت جراحی و اقدامات تهاجمی قلبی به علت ارتباط مستقیم با خون بیمار، آزمایشهای مکرر و استفاده از فرآورده‌های خونی شانس انتقال بیماریهای منتقله توسط خون به دیگران قابل توجه می‌باشد. ویروس هپاتیت B,Cو ایدز از عوامل مهم و خطرناک برای ایجاد بیماری مزمن و کشنده در بیماران و کارکنان بهداشتی درمانی و نیز سایر بیماران می‌باشد که در سالهای اخیر مورد توجه کادردرمانی قرار گرفته است. این مطالعه با هدف بررسی اپیدمیولوژیک موارد مثبتHBsAg،HCVAb،HIVAb در بیماران مورد جراحی و اقدامات تهاجمی قلبی انجام شد. روش بررسی:در این مطالعه توصیفی کلیه بیمارانی که جهت جراحی و اقدامات تهاجمی قلبی از سال 1387 تا سال 1391 در بیمارستان افشار یزد بستری شده بودند مورد بررسی قرار گرفتند. اطلاعات مورد نیاز از پرونده‌های بیماران استخراج گردید و با استفاده ازآمار توصیفی و آزمونChi-squareو نرم افزار16 SPSS مورد ارزیابی قرار گرفت. یافته ها: از 2414 بیمار بستری شده طی سالهای مورد مطالعه، 27 بیمار (1/1%) آلودگی HBV و 29 بیمار (2/1%) آلودگی HCV داشتند، هیچ مورد مثبتHIV مشاهده نشد. در مورد HBV بیشترین شیوع در محدوده سنی 16 تا 30 سال بود.در مورد HCV ارتباط معنی داری بین گروه های مختلف از نظر سن ، جنس و موقعیت جغرافیایی وجود داشت. به طوری که بیشترین شیوع در گروه سنی 31 تا 45 سال و در مردان2% و در زنان 1/0% بود (00/0P= ) و نیز آلودگی در افراد یزدی(7/1%) به طور معنی داری بیشتر از افراد غیر یزدی(3/0%) بود (003/0P= ). نتیجه گیری:شیوع هپاتیت در بیماران قلبی مشابه دیگر اقشار عموم مردم می‌باشد و لذا ضرورت انجام آزمایشات HBsAg،HCVAb،HIVAb قبل از عمل قابل تأمل است. پیشنهاد می‌شود انجام آزمایشات فوق در بیماران با احتمال آلودگی بیشتر مورد توجه قرار گیرد. واژه های کلیدی:هپاتیتB ،هپاتیتC،HIV،جراحی و اقدامات تهاجمی قلبی این مقاله حاصل از پایان نامه دکتری پزشکی دانشگاه علوم پزشکی شهید صدوقی یزد می باشد.

Title: بررسی رابطه فرسودگی شغلی با سلامت عمومی درکارمندان بانک استان گلستان در سال 91

http://tbj.ssu.ac.ir/article-1-1562-fa.html

مقدمه: فرسودگی شغلی نوعی از فرسودگی روانی است که با فشارهای روانی یا استرسهای مربوط به شغل و محیط کار توام گشته است و می تواند سلامت عمومی فرد را تحت تاثیر قرار دهد و بیشتر در شغل هایی که مرتبط با خدمات انسانی می باشد به وجود می آید، با توجه به مشکلات ویژه کارکنان بانک ها به دلیل حساسیت بالای کاری مطالعه حاضر به منظور بررسی رابطه فرسودگی شغلی با سلامت عمومی درکارمندان بانک استان گلستان انجام شد. روش بررسی: این مطالعه توصیفی – تحلیلی از نوع همبستگی می باشد. به منظور اهداف پژوهش روی 125کارمند بانک های دولتی از شهرستان مینودشت که به صورت سرشماری انتخاب شدند، انجام گرفت ابزار مورد استفاده شامل متغیرهای دموگرافیک، پرسشنامه سلامت عمومی -28سوالی و پرسشنامه فرسودگی شغلی مزسلاچ بود. پس از جمع آوری پرسشنامه ها داده ها وارد نرم افزار18 spss شده وآزمون های آماری T-Test،ANOVA ، ضریب همبستگی، 2χ در سطح 05/0 مورد تجزیه و تحلیل قرار گرفت. یافته ها: یافته ها نشان داد، در خصوص ابعاد مختلف فرسودگی شغلی خستگی عاطفی و مسخ شخصیت درسطح خفیف و عدم کفایت شخصی در سطح بالا قرار داشتند و از نظر سطح کلی فرسودگی شغلی در سطح متوسط بودند. بین سلامت عمومی و فرسودگی شغلی رابطه معنادار قوی معکوس وجود دارد. نتیجه گیری: براساس یافته های مطالعه، فرسودگی شغلی سلامت عمومی را تحت تاثیر قرار می دهد. از یافته های این مطالعه در برنامه ریزی پیشگیری، شناسایی گروههای در معرض خطر در محیط های کاری می توان استفاده کرد.

Title: بررسی تولید پسماند شهری در مناطق 22 گانه شهر تهران با رویکرد کاهش پسماند طی سال های 1392-1389

http://tbj.ssu.ac.ir/article-1-1555-fa.html

مقدمه:کاهش پسماند یک فاکتور کلیدی و زیرساختی اساسی در ایجاد جامعه پایدار است. اگرچه در سال های اخیر شهرداری تهران اقدامات خوبی در زمینه های مختلف مدیریت پسماند مانند جداسازی از مبدا، جمع آوری مکانیزه و احداث کارخانه های کمپوست انجام داده است، ولی میزان تولید پسماند در شهر تهران نه تنها روند کاهشی نداشته است بلکه افزایش نیز یافته است. در این مقاله ابتدا به وضعیت موجود مدیریت پسماند در شهر تهران پرداخته شده است. سپس با توجه به اینکه در تبیین استراتژی ها و برنامه‌ریزی‌های مرتبط با کاهش پسماند شناخت نوع و میزان پسماندهای تولیدی از اهمیت بسزایی برخوردار است، وضعیت تولید پسماند شهری در مناطق 22 گانه شهر تهران مورد ارزیابی قرار گرفته است. روش بررسی: این پژوهش یک مطالعه توصیفی مقطعی بوده که طی سال های 1389 تا 1392 با مراجعه به مسئولین بازیافت مناطق 22 گانه شهر تهران با تناوب سالیانه و با هدف جمع آوری اطلاعات موجود در خصوص تولید پسماند شهری مناطق صورت گرفت. مصاحبه حضوری با مسئولین سازمان مدیریت پسماند شهر تهران و تکمیل پرسشنامه، بازدید میدانی از مجتمع دفن و پردازش آرادکوه و جمع آوری اطلاعات در زمینه پسماندهای پذیرش شده توسط این مجتمع و سرنوشت نهایی این پسماندها از دیگر اقدامات صورت گرفته می باشد. سپس اطلاعات گردآوری شده مورد تجزیه و تحلیل قرار گرفت. یافته ها: میزان تولید کل پسماندهای شهر تهران طی سال‌های 1389 تا 1392 به ترتیب 3389662 ، 3399344 ، 3449338 و 3245157 تن بوده است که در سه گروه کلی پسماندهای شهری، پسماندهای شرکت ها و شهرک ها و پسماندهای بیمارستانی تقسیم بندی شده است. در این میان اکثر پسماند تولیدی شهر تهران مربوط به پسماندهای شهری شامل پسماندهای خانگی و تجاری مناطق 22 گانه تهران می باشد. با توجه به آخرین اطلاعات جمعیتی مناطق مختلف شهرداری تهران براساس سرشماری نفوس و مسکن که در سال 1390توسط مرکز آمار ایران به انجام رسیده است و طبق بررسی های انجام شده، سرانه تولید پسماند در 11 منطقه شهرداری تهران بین 550 تا 1000 و در 11 منطقه دیگر 1000 تا 1521 گرم به ازای هر نفر در روز در سال 1390 بوده است. کمترین سرانه پسماند تولیدی متعلق به منطقه 13 با 556 و بیشترین آن متعلق به منطقه 12 با 1521 گرم به ازای هر نفر در روز در سال 1390 بوده است. نتیجه گیری: مقایسه میزان سرانه تولید پسماند شهری در مناطق مختلف شهر تهران با حداکثر ظرفیت قابل قبول تولید پسماند، بیانگر فاصله تولید پسماند همه مناطق شهر تهران از حداکثر مقدار مجاز است. بنابراین علاوه بر اینکه برنامه ریزی و انجام اقدامات راهبردی جهت کاهش پسماند شهری تهران ضروری است، این برنامه ها و اقدامات باید با توجه به خصوصیات هر منطقه و کیفیت و کمیت پسماند آن منطقه طرح شوند.

Title: راهکارهای تغذیه ایی موثر بر پیشگیری از بروز سرطان معده

http://tbj.ssu.ac.ir/article-1-1570-fa.html

مقدمه: سرطان معده دومین علت مرگ و میر ناشی از سرطان در سراسر جهان بوده که مرگ و میر ناشی از آن در کشورهای آسیایی مانند ایران در حال افزایش می‫باشد. علیرغم استفاده از جراحی، اشعه درمانی و شیمی‫درمانی، میزان بقاء پنج ساله در بیماران در حدود 20 درصد می‫باشد. بنابراین درمان تکمیلی و پیشگیری از گسترش بیماری هدف اصلی در این زمینه می‫باشد. ما در این مقاله مروری برآنیم تا برخی راهکارهای موثر برای پیشگیری از وقوع سرطان معده را ارائه دهیم. روش بررسی: این مطالعه، مروری بر مقالات در مورد راهکارهای موثر برای پیشگیری از سرطان معده می باشد که در اینترنت و مجلات معتبر علمی جستجو شده است. با استفاده از کلید واژه های مناسب پایگاه های داده ای Google Scholar، Science Direct، Medline، Web of Science و Springer مورد بررسی قرار گرفتند. بحث و نتیجه گیری: مجموعه مطالعات بالینی با بکارگیری راه‌هایی که شانس وقوع و پیشرفت سرطان را به تأخیر می‌اندازد، جزو برنامه‌های پیشگیری قرار می‌گیرند. تحقیقات زیادی نشان داده اند که در سراسر دنیا خطر سرطان معده رابطه‏ای معکوس با شرایط اجتماعی، اقتصادی و تغذیه ای افراد دارد. یکی از مهمترین راهکارهای پیشنهادی پیشگیری کننده از سرطان معده استفاده از برنامه تغذیه مناسب، غنی از آنتی‏اکسیدان‏ها می باشد. خطر سرطان معده در افرادی با سبک زندگی سالم که دارای رژیم غذایی غنی از میوه‏ها و سبزیجات می‏باشند، کمتر است ولی استفاده از سیگار و تغذیه نامناسب با افزایش خطر بروز سرطان معده ارتباط دارند.

Title: بررسی اثربخشی آموزش بهداشت خواب بر بهبود اختلالات خواب پرستاران بیمارستان شهید صدوقی یزد

http://tbj.ssu.ac.ir/article-1-1556-fa.html

مقدمه: پرستاران به علت شب‌کاری مستعد اختلال خواب هستند. به منظور پیشگیری از خطاهای حرفه‌ای، تعالیم مفاهیم آموزش بهداشت خواب به پرستاران پیشنهاد می شود. هدف این مطالعه بررسی اثر بخشی آموزش بهداشت خواب بر بهبود اختلال خواب پرستاران می‌باشد تا منجر به تامین شرایط مطلوب کاری در بین این گروه گردد. روش بررسی: مطالعه نیمه تجربی حاضر به روش Field Trail با طرح after and before انجام شده است. داده‌ها با استفاده از پرسشنامه‌های دموگرافیک و(The Epworth Sleepiness Scale (ESS، SMII ( SleepMed Insomnia Index) و (Insomnia Severity Index) ISI جمع آوری شد. ابتدا پرسشنامه‌ها بین 207 نفر از پرستاران توزیع و تعداد صد100 نفر پرستار مبتلا به اختلال خواب شناسایی شد که پس از برگزاری 4 جلسه آموزشی 66 پرسشنامه با استفاده از نرم افزار SPSS مورد تحلیل قرار گرفت. یافته‌ها: یافته‌ها نشان داد که از 207 پرستار 3/48% دارای اختلال خواب بودند. همچنین مشخص شد که وضعیت اختلال خواب با فاکتورهایی نظیر سن، جنسیت، وضعیت تاهل، سابقه کاری، تعداد فرزندان، نوع نوبت کاری و رضایت شغلی ارتباط معنی‌دار ندارد. در بررسی رابطه آموزش بهداشت خواب با کاهش اختلال خواب مشخص شد که وضعیت اختلال خواب پرستاران بعد از دوره آموزشی بهبود یافته است که با توجه به 055/0 P= این ارتباط به طور نسبی معنی‌دار است. نتیجه گیری: با توجه به شیوع بالای اختلال خواب در پرستاران، عوامل دیگری همچون ویژگیهای ساختمانی محیط بیمارستان و اختلالات روان پزشکی نظیر افسردگی و اضطراب نیز باید مورد بررسی قرار گیرد. ضمناً آموزش بهداشت خواب به تنهایی موثر نبوده و باید در ترکیب با سایر روش‌های درمان رفتاری-شناختی به کار گرفته شود.

Title: اثربخشی آموزش راهبردهای شناختی بر عزت نفس و رضایت از زندگی در دانشجویان دختر کمال‌گرا

http://tbj.ssu.ac.ir/article-1-1559-fa.html

مقدمه: مطالعات نشان داده اند که افراد کمال‌گرا از عزت نفس و رضایت از زندگی کمتری برخوردارند. از آن جایی که عزت‌نفس و رضایت از زندگی از مولفه های اصلی سلامت روان به شمار می‌روند و با توجه به این نکته که فکر، احساس و رفتار سه کارکرد انسانی مجزا نیستند بلکه هر سه با هم ارتباط متقابل دارند. لذا این مطالعه با هدف بررسی اثربخشی آموزش راهبردهای شناختی بر عزت نفس و رضایت از زندگی در دانشجویان دختر کمال‌گرا انجام شد. روش بررسی: مطالعه حاضر یک مطالعه تجربی است. حجم نمونه 60 نفر بود که از بین دانشجویان دختر کمال‌گرا خوابگاهی (دانشگاه آزاد اسلامی واحد یزد) در سال تحصیلی 91 انتخاب شدند و به طور تصادفی در دو گروه آزمایش و کنترل قرار گرفتند. آموزش راهبردهای شناختی به صورت گروهی، برای گروه آزمایش اجرا شد. پس از گذشت دو ماه داده های حاصل از پژوهش با استفاده از آمار توصیفی و استنباطی مورد تجزیه و تحلیل قرار گرفت. یافته ها: نتایج نشان داد که عزت نفس در گروه آزمایش در مقایسه با پیش آزمون و گروه کنترل به طور معناداری افزایش (001/0>p) داشته، همینطور رضایت از زندگی نیز افزایش (001/0> p) معناداری را نشان می‌دهد و این نتایج در دوره پیگیری هم چنان پایدار بوده است. نتیجه گیری: با توجه به ثمر بخش بودن استفاده از راهبردهای شناختی در افزایش عزت نفس و رضایت از زندگی ، آموزش این راهبردها باید در راستای بازسازی شناختی، اصلاح الگوهای باور و تفکر غیرمنطقی و در نهایت بالا بردن سلامت روان صورت گیرد.

Title: تأثیر هشت هفته تمرین یوگا بر روی شاخص های تنفسی و ضربان قلب زنان غیرورزشکار

http://tbj.ssu.ac.ir/article-1-1558-fa.html

چکیده مقدمه: یوگا به عنوان نوعی ورزش برای بهبود وضع سلامت جسمی، روانی و اجتماعی و ارتقا احساس سلامتی افراد سالم در آموزش های پزشکی جامعه نگر مد نظر قرار گرفته است. از این رو مطالعه حاضر با هدف بررسی تأثیر هشت هفته تمرین یوگا بر روی شاخص های تنفسی و ضربان قلب زنان غیرورزشکار انجام شد. روش بررسی: دریک تحقیق تجربی از نوع قبل و بعد تعداد 30 نفر از دانشجویان به طور تصادفی انتخاب و به دو گروه 15 نفری شاهد و تجربی تقسیم شدند. قبل از شروع دوره تمرینات منتخب یوگا، شاخص های تنفسی که شامل: ظرفیت های حیاتی، ظرفیتهای ریوی، ظرفیت های تهویه اختیاری و تهویه دقیقه ای بود، توسط دستگاه اسپیرومتری مشخص شد، همچنین شمارش ضربان قلب در حالت استراحت از آزمودنیها به عمل آمد و پس از دو ماه انجام تمرینات یوگا که شامل تمرینات یوگا و تمرینات تنفسی(پرانایاما) می شد، مجدداً از کلیه آزمودنیها شاخص های تنفسی و ضربان قلب تحت شرایط مشابه قبلی اندازه گیری شد. داده ها با استفاده از آزمون آماری t تجزیه و تحلیل شد. یافته ها: نتایج نشان داد تعداد تنفس و ضربان قلب در حالت استراحت در گروه تجربی از لحاظ آماری کاهش معناداری داشت ولی میزان حجم بازدمی با فشار در ثانیه اول از لحاظ آماری در این گروه افزایش معناداری داشت، همچنین در پارامترهای ظرفیت حیاتی، حداکثر جریان بازدمی، حداکثر جریان دمی و حجم ریوی تغییری ایجاد نشد. نتیجه گیری: نتیجه تحقیق حاضر نشان داد 8 هفته تمرین یوگا باعث افزایش ظرفیت حیاتی و میزان حجم بازدمی با فشار در ثانیه اول و همچنین باعث کاهش تعداد ضربان قلب و تعداد تنفس در حالت استراحت می شود. واژه های کلیدی: یوگا ، پرانایاما، شاخص های اسپیرومتری، ضربان قلب

Title: سنجش رابطه بین معنویت در کار و تعهدسازمانی کارکنان اداری دانشگاه علوم پزشکی زاهدان

http://tbj.ssu.ac.ir/article-1-1560-fa.html

چکیده مقدمه: یکی از عوامل پنهان، ولی تأثیرگذار در رفتار شغلی کارکنان، میزان تعهد آنها به سازمان و شغل شان است. از این رو شناسایی عوامل تاثیرگذار بر تعهدسازمانی از وظایف مهم مدیران سازمانی می باشد که در مطالعه حاضر به نقش معنویت در کار در بروز آن پرداخته شده است. روش بررسی: این مطالعه توصیفی-همبستگی بوده که به صورت نمونه گیری تصادفی ساده و بر اساس فرمول نمونه گیری کوکران تعداد 151 نفر از کارکنان اداری دانشگاه علوم پزشکی زاهدان در آذرماه 1392 از طریق 2 پرسشنامه معنویت در کار میلیمن و دیگران و تعهدسازمانی لینز مورد مطالعه قرار گرفتند. برای تجزیه و تحلیل داده ها از ضریب همبستگی پیرسون و رگرسیون چندگانه با کمک نرم افزار 17SPSS استفاده شد. یافته ها: بین معنویت در کار و سه مولفه آن (معناداری کار، احساس همبستگی و همسویی ارزشها) با تعهدسازمانی رابطه مثبت و معنی داری وجود داشت (01/0p<). هم چنین هر سه مولفه معنویت در کار توانایی پیش بینی تعهدسازمانی را داشتند(05/0p<). نتیجه گیری: چنانچه معنویت در کار حاکم باشد و افراد کار خود را معنادار تلقی کنند، با دیگران احساس همبستگی و نوع دوستی داشته باشند و ارزش های فردیشان با ارزشهای سازمانی همسو و هماهنگ باشد تعهدسازمانی بیشتری به شغل و سازمان خود احساس خواهند نمود.

Title: ارزیابی وضعیت کیفیت خدمات، بر اساس رویکرد مدیریت ارتباط با بیمار (مورد مطالعه: بیمارستان خصوصی یزد)

http://tbj.ssu.ac.ir/article-1-1561-fa.html

مقدمه: افزایش تعداد مراکز ارائه خدمات درمانی، رقابت بر سر جذب بیمار را افزایش داده به طوری که بیمارستان‌ها با درک این موضوع اقدام به پیاده‌سازی مدل‌هایی برای افزایش رضایت بیمار کرده‌اند. امروزه بیمارستان‌ها برای افزایش رضایت بیماران علاوه بر ارائه‌ خدمات و محیط مراقبتی مطلوب، به گسترش روابط نزدیک و مدیریت ارتباط با بیمار (Patient Relationship Management) نیازمند می‌باشند. هدف از این تحقیق ارزیابی وضعیت کیفیت خدمات، بر اساس رویکرد PRM دریکی از بیمارستان های خصوصی یزد می‌باشد. روش بررسی: این تحقیق پیمایشی و توصیفی- اکتشافی می‌باشد. جامعه‌ آماری این تحقیق را بیماران بستری شده در بیمارستان خصوصی یزد تشکیل می‌دادند که بر اساس فرمول کوکران از بین آن‌ها 192 بیمار به عنوان نمونه انتخاب شدند. از آن جهت که تا کنون در زمینه‌ PRM تحقیقی صورت نگرفته ابزار ارزیابی مدیریت ارتباط با بیمار، پرسش‌نامه‌ای محقق‌ساخته با طیف پنج‌تایی لیکرت ( از خیلی ضعیف تا خیلی خوب) بود که روایی آن با استفاده از تحلیل عاملی و پایایی آن با استفاده از آلفای کرونباخ، مورد تأیید قرار گرفت. یافته‌ها: نتایج تحقیق حاکی از آن است که بیمارستان مورد مطالعه در تمام ابعاد به جز بُعد مشتری‌مداری پایین‌تر از سطح انتظار، عمل کرده و از بین 41 مولفه مورد بررسی تنها در 16 مولفه دارای وضعیت نامطلوب می‌باشد که از طریق ماتریس اهمیت- عملکرد (importance-performance matrix) اولویت رسیدگی به آن‌ها مشخص شده است. نتیجه‌گیری: به نظر می‌رسد بیمارستان مذکور با توجه به محدودیت‌‌های منابع و تلاش در جهت ارتقای کیفیت خدمات بیمارستانی خود بهتر است اولویت خود را "درک صحیح نیازهای کلیدی بیماران"، "تغییر در خدمات بیمارستان‌ در راستای نفع بیشتر بیماران"، "به کارگیری نظرات بیماران در طراحی خدمات"، "انعطاف پذیری مدیریت و کارکنان بیمارستان برای ارائه خدمات جدید"،" شناخت دقیق اطلاعات بیماران" و "ایجاد سیستم بازخورد دقیق" قرار دهد.

Title: اثردرمانی عصاره زنگوله ای لوله باریک برزخم سالک در موش BALB/C مبتلابه سالک

http://tbj.ssu.ac.ir/article-1-1564-fa.html

مقدمه: لیشمانیوز بیماری انگلی مشترک بین انسان و حیوان است که توسط گونه های مختلف انگل لیشمانیا ایجاد می شود ودر مناطق گرمسیر و نیمه گرمسیر جهان بومی است. یکی از شایع ترین اشکال این بیماری فرم پوستی آن است که متاسفانه از سراسر ایران به صورت همه گیر و بومی گزارش می شود، گیاه علفی کرک دار چند ساله به نام محلی "خوچوبه" و رسمی زنگوله ای لوله باریک " از خانواده گل گاوزبان و با نام علمی Onosma stinosiphon “. در گذشته به عنوان ضماد برای درمان بریدگی های سطحی پوست ،زخم و جوش مورد استفاده قرار گرفته است. هرچند که خواص درمانی این گیاه در هیچ کتاب دارویی ذکر نشده اما خاصیت ضدعفونی کننده گی اش در محل زخم به اثبات رسیده است، افزون بر اینکه عوارض ناخواسته جانبی ندارد. لذا هدف از این پژوهش تاثیرعصاره زنگوله ای لوله باریک برزخم سالک در موش BALB/C مبتلابه سالک می باشد. روش بررسی: این پژوهش از نوع تجربی و به شیوه کارآزمایی آزمایشگاهی است. انگل لیشمانیا گونه Leishmania tropica عامل سالک شهری به میزان مناسب در محیط های NNN و RPMI1640 کشت داده شد سپس موش ها در پنج گروه 10 تایی بشرح ذیل دسته بندی شدند.گروه های اول،دوم و سوم دریافت کننده غلظت های 80، 60 و 40 درصد عصاره گیاهی،گروه چهارم گروه کنترل بود. همه گروه ها همزمان به انگل مبتلا شدند. سپس ریشه گیاه زنگوله ای لوله باریک به میزان لازم تهیه و عصاره الکلی آن تهیه شد بلافاصله پس از ظهورزخم سالک در موش ها، پایش با اندازه گیری وزن موش و قطر زخم ها همراه با تجویز یک روز در میان غلضت های عصاره آغاز شد این کار تا زمان مرگ آخرین موش گروه کنترل ادامه یافت که روز تلف شدن موش های هر گروه یادداشت،ضمنا با تشریح موشها و پهنای طحال هر موش با خط کش اندازه گیری و سرانجام داده ها در رایانه ثبت و با استفاده از بسته نرم افزاریُSPSS مورد تجزیه و تحلیل قرار گرفت. یافته ها: میانگین وزن موشهای دریافت غلظت های متفاوت زنگوله ای لوله باریک با میانگین وزن موشهای شاهد اختلاف معنی دار نشان نداد (05/

Title: بررسی کارایی نانولوله کربنی چند جداره اکسید شده در حذف سرب از محیط آبی

http://tbj.ssu.ac.ir/article-1-1565-fa.html

مقدمه: نانولوله های کربنی موادی با قابلیت حذف برخی از آلاینده های آب هستند. کارایی این مواد در حذف آلاینده ها بسته به عوامل مختلفی از جمله PH، غلظت، زمان تماس و ... است.در تحقیق حاضر کارایی نانولوله کربنی چند جداره اکسید شده در حذف سرب از محیط های آبی مورد بررسی قرار گرفت. روش بررسی: مطالعه تجربی است. ابتدا نانولوله کربنی چند جداره اکسید شد و سپس در سه PH 4، 7 و 10 و زمانهای تماس 5، 10 و 40 دقیقه و غلظتهای 50، 100 و 125 میلی گرم بر لیتر نانولوله کربنی چند جداره اکسید شده، کارایی حذف سرب از محیط آبی مورد بررسی قرار گرفت.کلیه آزمایشات بر اساس روشهای مندرج در کتاب روشهای استاندارد برای آزمایشات آب و فاضلاب ویرایش بیست و یکم انجام شد. آزمایش ها تحت شرایط بهینه بر روی نمونه واقعی، آب آشامیدنی روستای ابراهیم آباد رضوی سیرجان انجام شد. تجزیه و تحلیل داده ها با استفاده از نرم افزار آماری SPSS نسخه 16 انجام گرفت. یافته ها: تغییرات همزمان زمان تماس و pH باعث تغییر کارآیی حذف سرب توسط نانولوله کربن چند جداره اکسید شده گردید. مهمترین عامل درافزایش راندمان حذف، استفاده از pH اسیدی برابر4 است و با افزایش همزمان زمان تماس و غلظت نانولوله، راندمان حذف افزایش داشت. در شرایط بهینه 125 میلی گرم بر لیتر غلظت نانولوله، زمان تماس 10 دقیقه و 4PH= راندمان حذف سرب در نمونه سنتتیک و نمونه واقعی به ترتیب 1/99 و 94 درصد حاصل شد. در مجموع اختلاف ناچیزی بین راندمان حذف درشرایط سنتتیک و واقعی وجود دارد که این اختلاف ناشی از تداخل کاتیونها، آنیونها و فلزات سنگین موجود در نمونه واقعی است. نتیجه گیری: نانولوله های کربنی چند جداره اکسید شده توانایی بالایی در حذف سرب از محیط های آبی دارند. بیشترین راندمان حذف در 4 PH= و کمترین راندمان حذف در 7PH= بوده است، لذا در شرایط اسیدی حذف با راندمان بالاتری انجام شده است.

Title: شناسایی عوامل موثر بر فضیلت گرایی و رتبه بندی آن عوامل در بیمارستان های آموزشی شهر یزد

http://tbj.ssu.ac.ir/article-1-1566-fa.html

چکیده مقدمه:در چند دهه اخیر سازمان‌ها گرایش بیشتری به سمت فضائل اخلاقی و انسانی پیدا کرده‌اند و از این طریق سعی نموده‌اند تا خلاءهای معنوی و اخلاقی و عاطفی کارکنان خود را بر طرف سازند. هدف از این پژوهش، شناسایی عوامل موثر بر فضیلت گرایی در بیمارستان های آموزشی شهر یزد و رتبه بندی این عوامل می باشد. روش بررسی: تحقیق حاضر از نوع تحلیلی بوده و به صورت مقطعی و کاربردی در شش ماهه دوم سال 1393 در بیمارستان های آموزشی شهر یزد انجام شد. جامعه آماری کلیه کارکنان بیمارستان های آموزشی شهر یزد بودند.روش نمونه گیری، تصادفی ساده بود و 184 پرسش نامه جمع آوری شد. داده ها با استفاده از پرسش نامه بسته شامل 56 سوال که روایی و پایایی آن بررسی شده بود، جمع آوری گردید. تحلیل داده ها با استفاده از نرم افزار آماری 18 SPSS صورت گرفت. برای استخراج عوامل موثر بر فضیلت گرایی از آزمون های تحلیل عاملی اکتشافی و تحلیل عاملی تاییدی و برای رتبه بندی عوامل از آزمون فرید من استفاده شد. یافته ها: نتایج تحلیل عاملی اکتشافی نشان داد که عوامل موثر بر فضیلت گرایی در قالب شش عامل مدیریت و رهبری، منابع انسانی، ساختار و فرایند، فرهنگ سازمانی، توجه به جامعه و چشم انداز قابل تبیین هستند. نتیجه گیری: عوامل فرهنگ سازمانی و توجه به جامعه و چشم انداز نسبت به عوامل مدیریت و رهبری، ساختار و فرایند و منابع انسانی اولویت پایین تری دارد. برای ارتقاء این عوامل و نهایتا ارتقاء سطح فضیلت محوری در بیمارستان ها پیشنهاد می شود شاخص های ارتقاء جو اخلاقی در سازمان، توجه به امور عام المنفعه و انسان دوستانه، نفوذ فضایل در استراتژی، اهداف و چشم انداز تقویت شوند.

Title: نقش واسطه ای جوخانواده درارتباط باسلامت عمومی دانش آموزان دختر دبیرستانهای شهر یزد

http://tbj.ssu.ac.ir/article-1-1567-fa.html

مقدمه:خاﻧﻮاده ﻣﺎﻧﻨﺪ ﻫـﺮ ﻧﻈﺎم اﺟﺘﻤﺎﻋﻲ دارای ﻧﻴﺎزﻫﺎی اوﻟﻴﻪ اﺳﺖ،از ﺟﻤﻠه می توان به اﺣﺴـﺎس ارزﺷـﻤﻨﺪی، اﻣﻨﻴـﺖ ﻓﻴﺰﻳﻜﻲ، ﺻﻤﻴﻤﻴﺖ،ﺗﻌﻠﻖ و واﺑﺴﺘﮕﻲ، اﺣﺴﺎس مسئولیت، ﻧﻴـﺎز ﺑﻪ اﻧﮕﻴﺰه و ﺗﺄ ﻳﻴﺪ دﻳﮕﺮان، ﻧﻴﺎز ﺑﻪ ﺷـﺎدی و ﻣﻮﻓﻘﻴـﺖ و ﻧﻴﺎزﻫـﺎی ﻣﻌﻨﻮی خانواده وبه خصوص دختران اﺷﺎره ﻛﺮد، ﻫـﺮ ﺧـﺎﻧﻮاده وﺿـﻌﻴﺖ و ﻓﻀﺎی ﻋﺎﻃﻔﻲ ﻣﻨﺤﺼﺮ ﺑﻪ ﻓﺮد داردو روش اداره و ﻧﻮع ﻋﻤﻠﻜـﺮد آنها ﺑــﺎ ﻳﻜــﺪﻳﮕﺮ ﻣﺘﻔــﺎوت است. اﮔﺮ اﻓﺮاد ﺟﺎﻣﻌﻪ ﺑﻪ وﻳﮋه واﻟﺪﻳﻦ ازتاﺛﻴﺮ ﺟﻮ ﻋﺎﻃﻔﻲ ﺧـﺎﻧﻮاده و ﻋﻤﻠﻜﺮد ﺧﻮد ﺑـﺮ روی ﺳـﻼﻣﺖ و ﺷـﻜﻮﻓﺎﻳﻲ ﻓﺮزﻧـﺪان آﮔـﺎه ﺑﺎﺷــﻨﺪ، ﺳــﻌﻲ ﺧﻮاﻫﻨــﺪ ﻛــﺮد ﻛــﻪ ﻓﻀــﺎﻳﻲآرام، ﻣﺘﻌــﺎدل و ﺣﻤﺎﻳﺖ ﻛﻨﻨﺪه را ﺑﺮای ﻓﺮزﻧﺪان ﺧﻮﻳﺶ ﻣﻬﻴـﺎ ﻛﻨﻨـﺪ. بنابراین بر آن شدیم که مطالعه ای در زمینه ارتباط سلامت عمومی دانش آموزان وجو خانواده را مورد بررسی قرار دهیم. روش بررسی: این پژوهش ازنوع توصیفی به روش پیمایشی می باشد جامعه آماری 374 نفراز دختران دانش آموز دبیرستانی شهر یزد بودندکه به صورت خوشه ای ازبین دانش آموزان دبیرستانهای یزد انتخاب شدند.اطلاعات مورد نیاز با پرسشنامه های سلامت عمومی( GHQ28 )و پرسشنامه جو خانواده جمع آوری گردید.داده ها با استفاده از نرم افزار SPSS نسخه16 وآزمونهای توصیفی وتحلیلی تجزیه وتحلیل گردید. یافته ها: نتایج این پژوهش نشان دادکه کلیه دانش آموزان از نظرمقیاسهای مختلف سلامت عمومی (مقیاس علائم جسمانی، مقیاس علاپم اضطرابی،مقیاس کارکرد اجتماعی ومقیاس علائم افسردگی) درحد خفیف بودند.داده ها بیان می کنند که بین تمام سازه های پرسشنامه جو خانواده با مقیاسهای سلامت عمومی به استثنای سازه ، بازبودن بادیگران، رابطه معنی دارومنفی (001/>p ) وجوددارد. بحث و نتیجه گیری:نتایج این بررسی ومقایسه آن با نتایج بررسی های دیگر راهنمای بسیارمناسبی برای آموزش وتربیت کودکان ودانش آموزان در خانواده ومدارس می باشد ووالدین می توانند ازآنها در تامین سلامتی کودکانشان استفاده نمایند.
